# Genome Sequences of a Staphylococcus aureus Clinical Isolate, Strain SMA0034-04 (UGA22), from Siaya County Referral Hospital in Siaya, Kenya

**DOI:** 10.1128/MRA.01627-18

**Published:** 2019-04-25

**Authors:** Gary Xie, Qiuying Cheng, Hajnalka Daligault, Karen Davenport, Cheryl Gleasner, Lindsey Jacobs, Jessica Kubicek-Sutherland, Tessa LeCuyer, Vincent Otieno, Evans Raballah, Harshini Mukundan, Douglas J. Perkins, Benjamin McMahon, Norman Doggett

**Affiliations:** a Biosecurity and Public Health, Bioscience Division, Los Alamos National Laboratory, Los Alamos, New Mexico, USA; b Center for Global Health, Department of Internal Medicine, University of New Mexico Health Sciences Center, Albuquerque, New Mexico, USA; c Theoretical Biology and Biophysics, Theoretical Division, Los Alamos National Laboratory, Los Alamos, New Mexico, USA; d Physical Chemistry and Applied Spectroscopy, Chemistry Division, Los Alamos National Laboratory, Los Alamos, New Mexico, USA; e University of New Mexico Laboratories of Parasitic and Viral Diseases, Kisumu, Kenya; f Department of Medical Laboratory Sciences, School of Public Health, Biomedical Sciences and Technology, Masinde Muliro University of Science and Technology, Kakamega, Kenya; University of Maryland School of Medicine

## Abstract

Here, we report the genome sequences of a Staphylococcus aureus clinical isolate, strain SMA0034-04 (UGA22), which contains one chromosome and one plasmid. We also reveal that isolate SMA0034-04 (UGA22) contains loci in the genome that encode multiple exotoxins.

## ANNOUNCEMENT

Staphylococcus aureus, a Gram-positive, round-shaped Firmicutes bacterium, is a common cause of skin infections, including abscesses, respiratory infections (such as sinusitis), and food poisoning. Pathogenic strains often promote infections by producing virulence factors, such as potent protein toxins, and by the expression of a cell surface protein that binds and inactivates antibodies. We report here the draft genome sequences of S. aureus strain SMA0034-04 (UGA22), isolated from the venous blood of a febrile female pediatric patient (10.8 months old) at the Siaya County Referral Hospital in western Kenya in 2004. Diagnostic results revealed that the bacteremic patient was HIV negative but had Plasmodium falciparum malaria with thrombocytopenia.

Although S. aureus is a common nosocomial pathogen, this was not the case here. Prior to any treatment interventions, blood was collected upon admission into a pediatric 1.5-ml microbial tube (Isolator, Wampole Laboratories, Cranbury, NJ) and was cultivated at 35°C for 18 to 24 h in 5% CO_2_ on 5% sheep blood agar. Bacterial DNA was extracted from a pure culture using the UltraClean microbial DNA isolation kit (Qiagen, Germantown, MD) according to the manufacturer's instructions with minimal modifications. The library was prepared from 100 ng of bacterial DNA using a NEBNext Ultra DNA library prep kit for Illumina (New England Biolabs, Ipswich, MA). S. aureus strain SMA0034-04 DNA was draft sequenced to 122-fold coverage using a MiSeq v2 500-cycle sequencing kit (Illumina, San Diego, CA), resulting in 3,480,522 paired-end 251-bp reads. The reference genome and plasmid were determined by the top megablast search hit against the NCBI NR database. The Burrows-Wheeler Aligner (BWA) version 0.7.2 (1) was used to map SMA0034-04 reads covering 94.78% of the S. aureus subsp. aureus MW2 chromosome (2) and 100% of S. aureus pWBG745, a conjugative plasmid that has been known to mobilize unrelated antimicrobial resistance/virulence genes (3). Data quality was assessed and the data files were filtered and trimmed with FaQCs version 1.3 (4) and then assembled with Velvet version 1.2.08 (5, 6) and IDBA version 1.1.0 (7). The consensus sequences were computationally shredded and reassembled with Phrap version SPS-4.24 (8, 9) to allow some manual editing with Consed (10), resulting in a final 80 contigs (81.43% of the reads) of >200 bp, with an *N*_50_ value of 621,451 bp, for SMA0034-04. Among these, contig 71 (pUGA22) encodes the intact *rep24*-type plasmid of an *S. aureus* pWBG745 homolog. The draft genome sequence of SMA0034-04 consists of 2,608,371 bp, with an average G+C content of 32.9%. Annotations were completed at Los Alamos National Laboratory (LANL) using an automated system with the Ergatis workflow manager (11) and in-house scripts.

There are 2,512 predicted protein-coding genes, 26 tRNA genes, and 12 rRNA genes within the genome of SMA0034-04. Of these, 37% of the protein-coding genes were annotated in a SEED subsystem (12), whereas 63% were not associated with a SEED subsystem. A total of 740 genes were annotated as hypothetical proteins. Of all the predicted genes, 2,295 are shared between SMA0034-04 and the S. aureus subsp. *aureus* MW2 genome, with 217 and 273 genes being unique to SMA0034-04 and MW2, respectively. The plasmid pUGA22 shares 99.93% identity and 100% query coverage with its counterpart, pWBG745. The main differences are 4 hypothetical protein genes that are deleted in pUGA22 and that antibacterial resistance genes are absent in pUGA22. The estimated antibacterial resistance profile (13) is similar between SMA0034-04 and MW2, except for methicillin resistance genes in the MW2 chromosome. A genome survey of toxin and virulence factor genes revealed the presence of protein A and superantigen-like exotoxins (SSLs) on SMA0034-04 contig 78; the presence of exfoliative toxin and staphylococcal alpha and delta toxins on contig 80; and the presence of a gamma-hemolysin hlgACB cluster, Panton-Valentine leukocidin (PVL) cytotoxin, and extracellular metalloprotease aureolysin (AUR) on contig 79. The enterotoxin type B gene (*entB*) is truncated by a missense mutation on SMA0034-04 contig 79, and the beta-hemolysin (beta toxin) gene is interrupted by a phage element on contigs 74 and 80. This phage integrated into beta-hemolysin has been recorded in many S. aureus strains of human origin (14).

### Data availability.

The GenBank accession number for the Staphylococcus aureus SMA0034-04 (UGA22) genome sequences is NWTZ00000000. The raw reads have been deposited in the NCBI Sequence Read Archive under accession number SRR8648187.
